# The remodeling of Z-DNA in the mammalian germ line

**DOI:** 10.1042/BST20221015

**Published:** 2022-12-01

**Authors:** Yingying Meng, Piroska E. Szabó

**Affiliations:** Department of Epigenetics, Van Andel Institute, Grand Rapids, MI 49503, U.S.A.

**Keywords:** DNA methylation, epigenome remodeling, genome integrity, prospermatogonia, Z-DNA, ZBTB43

## Abstract

We recently discovered a novel biological process, the scheduled remodeling of Z-DNA structures in the developing fetal mouse male germ cells [Nat. Cell Biol. 24, 1141–1153]. This process affects purine/pyrimidine dinucleotide repeat (PPR) rich sequences, which can form stable left-handed Z-DNA structures. The protein that carries out this function is identified as ZBTB43, member of a large family of ZBTB proteins. Z-DNA remodeling by ZBTB43 not only coincides with global remodeling of DNA methylation and chromatin events in the male germ line, but it also is a prerequisite for *de novo* DNA methylation. When ZBTB43 changes DNA structure from the left-handed zigzag shaped Z-DNA to the regular smooth right-handed B-DNA, it also generates a suitable substrate for the *de novo* DNA methyltransferase, DNMT3A. By instructing *de novo* DNA methylation at PPRs in prospermatogonia, ZBTB43 safeguards epigenomic integrity of the male gamete. PPRs are fragile sequences, sites of large deletions and rearrangements in mammalian cells, and this fragility is thought to be due to Z-DNA structure formation rather than the sequence itself. This idea is now supported by the *in vivo* finding that DNA double strand breaks accumulate in mutant prospermatogonia which lack ZBTB43-dependent Z-DNA remodeling. If unrepaired, double stranded DNA breaks can lead to germ line mutations. Therefore, by preventing such breaks ZBTB43 is critical for guarding genome stability between generations. Here, we discuss the significance and implications of these findings in more detail.

## Global epigenome remodeling in the germ line

**Figure 1.**
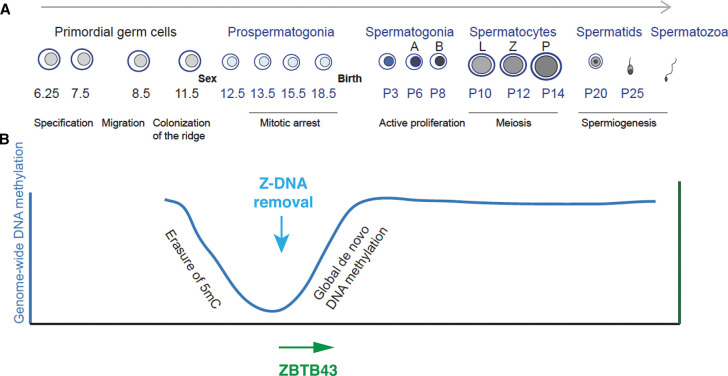
Remodeling Z-DNA by ZBTB43 coincides with global epigenome remodeling in the male germ line. (**A**) Schematic representation of the development of male germ cells in the mouse from the primordial germ cells of the embryo, prospermatogonia of the fetus, postnatal mitotic spermatogonia, meiotic leptotene (L), zygotene (Z) and pachytene (P) spermatocytes, round and elongating spermatids to adult spermatozoa. The age of the mouse is marked in days post coitum (dpc) before birth and in postnatal days (P3, P6, etc.) after birth. (**B**) Z-DNA removal occurs at the initiation of global *de novo* methylation in the male germ line. Global level of DNA methylation across developmental stages is depicted by the dark blue line. DNA methylation reaches the lowest level at 13.5–15.5 days post coitum (dpc) and a burst of *de novo* methylation takes place between 15.5–17.5 dpc, and methylation levels remain high until the spermatozoa stage. ZBTB43 expression and Z-DNA removal is detected at 15.5 dpc. ZBTB43 is required for removing Z-DNA structure at PPRs, as shown by immunohistochemistry of wild type and *Zbtb43*^−/−^ prospermatogonia.

Our recent study reveals a novel layer of global remodeling of the epigenome [1]. Chromatin composition includes covalent modifications of DNA and histone molecules. These marks can be faithfully maintained between cell divisions and provide the epigenetic memory, which instructs each cell type to display specific physical characteristics and to carry out specific functions. Epigenome changes may occur stepwise and globally. In mammalian species, two waves of global remodeling events take place, one at the soma-to germ line and the other at the germ line-to soma transitions. The mammalian germ line undergoes global epigenome remodeling at the level of DNA methylation and chromatin [2–9] to erase the somatic pattern, and to establish the male or female gamete-specific epigenomes. DNA methylation levels are high in the germ line at the emergence of primordial germ cells, but this pattern is erased in both sexes as the germ cells migrate and colonize the genital ridges. Female germ cells reestablish DNA methylation only after birth in the growing meiotic oocytes whereas male germ cell undergo global *de novo* methylation in the mitotically arrested fetal male germ cells (prospermatogonia) (Figure 1). The male gamete's epigenome largely relies on efficient events that take place in a time window of 2 days between 15.5–17.5 days post coitum (dpc) in the fetal germ cells. This global *de novo* methylation is carried out by *de novo* DNA methyltransferases DNMT3A and DNMT3C with the help of the catalytically inactive DNMT3L [10–13]. The pattern of DNA methylation is globally different in spermatozoa compared with primordial germ cells and also in spermatozoa compared with oocytes, suggesting that *de novo* methylation involves genomic location-specific instructions that lend such cell-specificity. Such instructions in prospermatogonia include piRNAs [14–16] and histone composition covalent modifications. Di- and trimethylation of H3K4 is restrictive to DNA methylation [3], whereas H3K36me2 is instructive [17] in prospermatogonia. We earlier detected broad low-level transcription along the genome in prospermatogonia [3], and we expect that this may facilitate the default *de novo* methylation at broad regions, potentially by instructing histone marks. This hypothesis is currently being tested genetically. In addition to the above mechanism, we explored the possibility that sequence-specific DNA-binding protein factors provide instructions to the DNA methylation machinery, and we considered the role of ZBTB proteins.

## ZBTB proteins

The family of broad-complex, Tramtrack and Bric-à-brac (BTB) — zinc finger domain containing (ZBTB) proteins [18] consists of 49 members which control various developmental and cellular processes [19–22]. Each ZBTB protein contains the BTB domain, which mediates protein–protein interactions and varying numbers of C2H2 zinc fingers (ZF), which can recognize and bind specific DNA sequences. The ZBTB protein family was considered for potentially guiding *de novo* DNA methylation, because some members of this family can distinguish 5-methylcytosine (^5m^C) from unmethylated C. For example, ZBTB2 preferentially binds unmethylated DNA, whereas ZBTB4 and ZBTB38 preferentially bind methylated DNA [18,23–27]. ZBTB33 (Kaiso) can also distinguish the methylated state of DNA, but its binding to methylated [28–30] or unmethylated DNA [31] may be context and protein conformation dependent [32–34]. ZBTB33 affects DNA methylation patterns in cell culture [35–37]. Another family member, ZBTB24 is implicated in the immunodeficiency, centromeric instability and facial anomalies 2 (ICF2) syndrome [38–42], a genetic disorder characterized by DNA hypomethylation among other features [43,44]. ZBTB24 binds DNA and regulates transcription of specific genes, such as CDCA7, but it is not clear how ZBTB24 affects DNA methylation [44–48].

## Alternative DNA structures

In addition to the common right-handed B-DNA helix, DNA can adopt alternative conformations, such as DNA hairpin, triplex DNA, G-quadruplex and Z-DNA [49–51]. Alternative DNA structure-forming sequences are frequently found at fragile sites in the mammalian genome, where genomic rearrangements, deletions and translocations occur, which are associated with human diseases [52]. Genomic sites that have the ability to form secondary structures exhibit double strand breaks (DSB) in human cells with risks to genome stability [53].

It is important to consider alternative DNA structures from the point of epigenome remodeling as well. Microsatellite-like purine/pyrimidine repeat sequences (PPRs), such as (CG)n, (CACG)n are prone to forming the left-handed zigzag shaped double helix, called Z-DNA [54]. Because its distinct shape, Z-DNA is not recognized as substrate for enzymes that modify B-DNA, such as bacterial DNA modification/restriction enzymes and the human DNA methyltransferase 1 (DNMT1) [55–57]. If PPRs existed in the Z-DNA structure in the germ cells that undergo epigenome remodeling, can they undergo *de novo* methylation?

**Figure 2.**
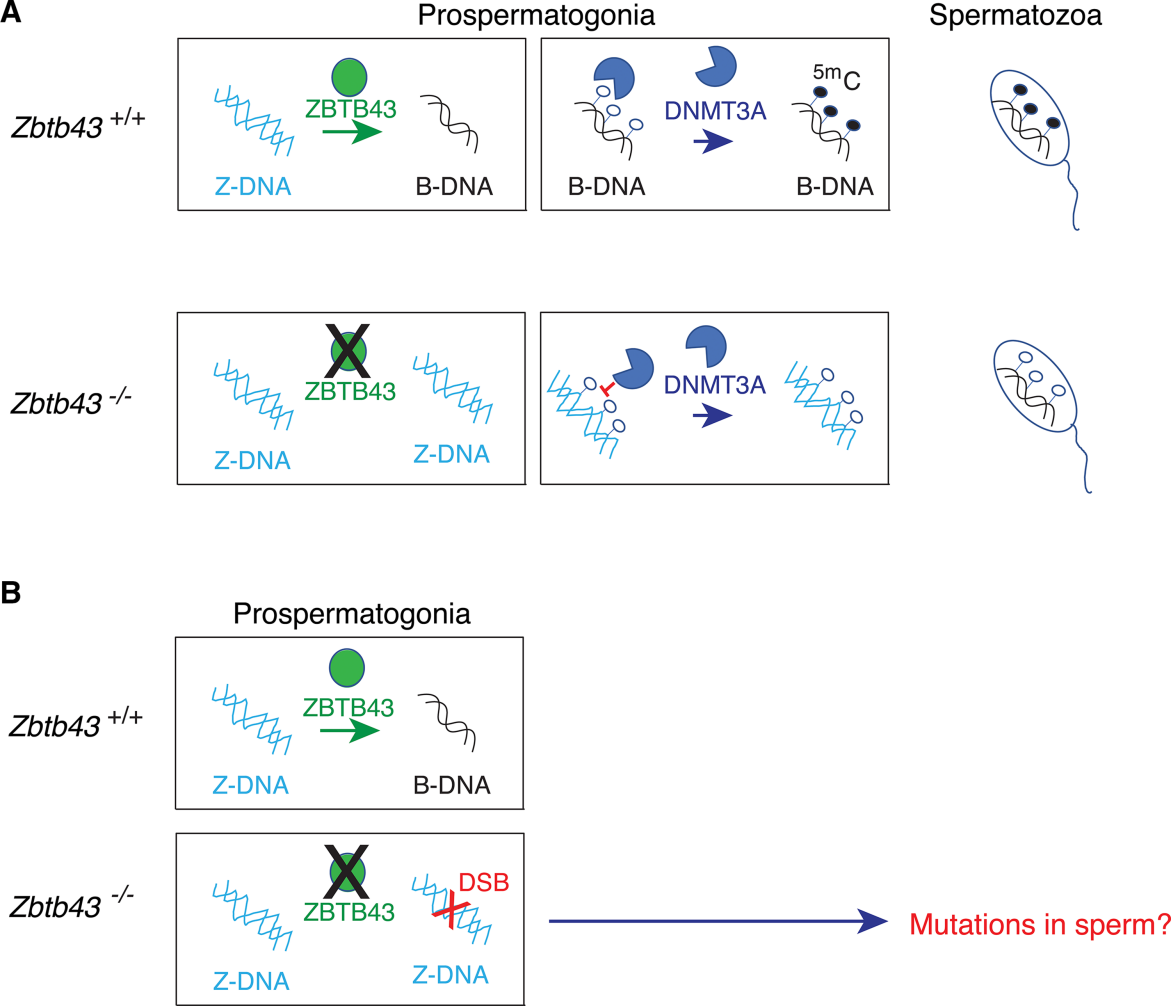
Removing the Z-DNA structures in propermatogonia has consequences to epigenome integrity and genome stability. (**A**) Effect on epigenome stability. ZBTB43 facilitates the process of *de novo* DNA methylation in the fetal male germ cells. Top row: In wild type prospermatogonia ZBTB43 binds at PPR sequences that exist in the Z-DNA structure and changes the DNA structure. By doing this, ZBTB43 creates B-DNA which can be recognized as substrate by methyltransferase enzymes. DNA methylation establishment occurs normally at PPR-rich DNA regions at the fetal stage, and CpG methylation persists through the spermatozoa stage. Bottom row: In the *Zbtb43*^−/−^ mutant prospermatogonia that lacks ZBTB43 protein, PPRs remain in the Z-DNA structure, and do not undergo *de novo* DNA methylation. Hypomethylation of PPRs is detected in prospermatogonia and sperm. (**B**) Effect on genome stability. Compared with the wild type cells (top row), *Zbtb43*^−/−^ mutant prospermatogonia (bottom row) exhibit speckles of γH2AX in immunohistochemical analysis, indicating that in the absence of ZBTB43 DNA double strand breaks occur due to the Z-DNA structure in these cells *in vivo*. These breaks may result in genomic deletions or rearrangements, because ZBTB43 binding sites are highly mutagenic in mammalian mutation assays.

We detected a novel biological process, the scheduled remodeling of Z-DNA structure in the organism that takes place in the developing fetal male germ cells and this remodeling of Z-DNA structure coincides with and instructs *de novo* methylation of the underlying microsatellite sequences [1] (Figure 1). Our work revealed that one previously uncharacterized member of the ZBTB protein family, ZBTB43 is sensitive to DNA methylation, and it also instructs *de novo* DNA methylation to specific sites in the genome [1]. Those sites can form Z-DNA structures, and ZBTB43 affects DNA methylation by remodeling the alternative DNA structure Z-DNA in prospermatogonia. This remodeling has consequences to the integrity of the sperm epigenome (Figure 2A) and to genome stability of the species (Figure 2B).

## ZBTB43 safeguards epigenome integrity

ZBTB43 is specifically and highly expressed in 15.5 dpc prospermatogonia at the onset of global *de novo* DNA methylation (Figure 1). ZBTB43 can bind both methylated and unmethylated DNA, with higher affinity to methylated DNA [1]. In addition, using genome-wide affinity-based MIRA-seq we found that spermatozoa in *Zbtb43^−/−^* mice exhibit methylation deficiency at over 300 PPR regions in the mouse genome. Using chemical bisulfite DNA sequencing we confirmed some of those differentially methylated regions (DMRs) in sperm DNA and found that they existed in late stage prospermatogonia already. This genetic approach identifies ZBTB43 as a unique DNA-binding factor required for specifying locations of *de novo* DNA methyltransferase activity at PPR sequences in prospermatogonia during the critical window of global epigenome remodeling in the mouse germ line (Figure 1).

Our investigation using biochemical approaches further revealed that ZBTB43 binds at PPRs directly and changes DNA structure from the left-handed Z-DNA to right-handed B-DNA, thus generating the substrate which can be methylated by DNMT enzymes, such as DNMT1 [55–57]. We found using immunochemistry using the Z-DNA antibody that the Z-DNA structure becomes less abundant globally in prospermatogonia between 13.5 dpc and 15.5 dpc (Figure 1). Using a genetic approach, we found that Z-DNA removal is an active process and depends on ZBTB43, because it does not take place in *Zbtb43^−/−^* fetal testis samples. Remodeling Z-DNA structure can now be counted among the major remodeling events in the germ line in addition to remodeling chromatin structure and DNA methylation (Figure 1). Importantly, remodeling of Z-DNA to B-DNA by ZBTB43 is, a prerequisite for *de novo* DNA methylation (Figure 2A). At PPRs ZBTB43 affects the function of DNMT3A, the *de novo* methyltransferase responsible carrying out *de novo* methylation in prospermatogonia [58]. This can be achieved indirectly by generating B-DNA at PPRs. Our biochemical experiments showed that, indeed, Z-DNA structures are not suitable substrates for DNMT3A. It is unlikely that ZBTB43 directly recruits DNMT3A to PPRs, as we found no protein–protein interaction between ZBTB43 and DNMT3A by co-immunoprecipitation experiments. Future genome-wide mapping experiments will determine the dynamics of ZBTB43 binding to PPRs, Z-DNA structure, and DNA methylation during prospermatogonia development at specific PPRs *in vivo*. We expect to find that Z-DNA is lost in prospermatogonia at specific PPRs after ZBTB43 binding has occurred at that site, and DNA methylation is established at PPRs after Z-DNA is removed. ZBTB43 may dissociate from the PPRs after remodeling Z-DNA structure, and this can explain the relatively small number of *in vivo* ChIP-seq peaks compared with *in vitro* affinity-seq peaks. On the other hand, the dissociation of ZBTB43 from PPRs may not be an immediate reaction, because ZBTB43 can bind to PPRs in the B-DNA form as well, consistent with detecting ZBTB43 ChIP-seq peaks in 15.5 dpc. prospermatogonia, which have largely undergone the Z-DNA to B-DNA remodeling. ZBTB43 may remain bound to PPRs after they become methylated, this would be consistent with the preference of ZBTB43 to methylated DNA over unmethylated DNA *in vitro*. Because DNA methylation is known to induce Z-DNA [59,60], ZBTB43 could have an important role in preventing re-formation of Z-DNA in the late-stage prospermatogonia.

## ZBTB43 safeguards genome integrity

The integrity of the male gamete's epigenome as we described above (Figure 2A), and the genome integrity of the species (Figure 2B) both require remodeling Z-DNA by ZBTB43. PPRs are risk factors for DNA translocations in cancer [61,62]. It has been shown that PPRs induce DSBs in mammalian cells [63–65]. (CG)n repeats form Z-DNA structures, and these are recognized and cleaved by ERCC1-XPF and other enzymes, producing DSBs [63]. The fragility of PPRs is thought to be due to Z-DNA formation, but this idea has not been formally proven, because there hasn't been a way to manipulate Z-DNA structure in the organism *in vivo*. Our genetic evidence gives support to this idea: we detected γH2AX speckles indicating double-strand break formation in prospermatogonia of *Zbtb43^−/−^* fetal testis samples [1]. This finding suggests that PPRs break because they exist in the Z-DNA structure in the *Zbtb43^−/−^* mutant fetal germ cells in the absence of scheduled remodeling by ZBTB43. Because ZBTB43 is most robustly detected at the perinuclear regions, γH2AX signals would be expected at these regions in the mutant prospermatogonia, but they appear more centrally. We speculate that one role of ZBTB43 might be to recruit and retain the PPR DNA at the nuclear periphery where those microsatellites would be protected from breakage. In the absence of ZBTB43, those mutagenic sites would be released into more central positions of the nucleus where the breaks could then occur more easily. Follow-up genome-wide mapping experiments will determine the dynamics of DNA structural changes from Z-DNA to B-DNA. We expect to find Z-DNA and DSB formation in *Zbtb43^−/−^* mutant prospermatogonia at the PPR sites where ZBTB43 binding is detected in normal prospermatogonia.

One prominent PPR-rich ZBTB43-binding peak from an intron of *Rps6kl1* was found highly mutagenic in mammalian COS-7 cells [1]. Mutagenicity of this site could even be higher in Zbtb43*^−/−^* prospermatogonia, which are mitotically arrested cells, and DSBs at Z-DNA structures could not be repaired by error-free homologous recombination. When DSBs are repaired through error-prone DSB repair mechanisms, such as the nonhomologous DNA end joining (NHEJ) [66,67] or the microhomology-mediated end joining (MMEJ) repair pathways [68,69], large-scale genomic rearrangements, deletions and translocations occur in a replication-independent manner [63,64,70,71]. If such mutagenic events take place in the soma, they might lead to disease, such as cancer. However, if such mutations arise in the germ line, they might endanger the genome integrity of the species, or impact genome evolution [65,72]. If the *Rps6kl1* site, for example, broke and was repaired by the NHEJ or MMEJJ pathway, this could lead to germ line mutations. *In vitro* assays showed that ZBTB43 binds this sequence in the Z-DNA structure and changes it to B-DNA. ZBTB43 facilitates *de novo* DNA methylation at this site in prospermatogonia, which suggests that ZBTB43 binds and remodels this DNA fragment into B-DNA *in vivo*. By eliminating the Z-DNA structures in the mouse germ line, ZBTB43 lessens the risk of DSBs and safeguards genome stability of the species.

It will be important to find out whether the DSB formation in the *Zbtb43^−/−^* prospermatogonia leads to mutations, which then could be passed to the next generation via the sperm. Future whole genome sequencing experiments of single sperm DNA will answer this question. We do not have the evolutionary time at hand to isolate advantageous mutations which will be present in each cell of the offspring from the *Zbtb43* mutant germ line. Nature, however, has carried out such an experiment in the Stickleback fish [65]. Sticklebacks lack the gene that encodes the ZBTB43 protein, and in its germ cells Z-DNA would not be expected to be remodeled at PPRs, and the fragile Z-DNA would be expected to break frequently. Repeated loss of pelvic structures during evolution was observed in pairs of Stickleback species. Loss of pelvic fins lent an advantage to the lake fish, compared with the sea water fish, and it was selected for during parallel evolution. This anatomical change corresponded in each case to a genomic deletion of an enhancer at a PPR-rich region. This PPR rich region was shown to be mutagenic in COS-7 cells, and to be able to adopt the Z-DNA structure *in vitro*, similar to the PPR-rich region that binds ZBTB43 in the mouse germ cells. It remains to be shown that the Stickleback fragile site forms Z-DNA and in the germ cells.

## Outlook and open questions

ZBTB43 has a role in the growth and perinatal viability in the mouse, according to the parental effect phenotypes observed in our mouse breeding experiments. This intergenerational effect from germ line to offspring may be related to regulating Z-DNA in prospermatogonia at specific PPR-rich regions in the genome. Future experiments will answer whether this involves one or both ZBTB43 functions, safeguarding epigenome and genome integrity in the germ line. It is possible that the partially penetrant parental effect phenotypes are the result of aberrant DNA methylation establishment in the germ line, which is passed on to the offspring, and may not fully be maintained. It is less likely that germ line mutations occur at fragile PPR regions with high enough frequency to affect 50–60% of the offspring out of *Zbtb43^−/−^* parents. Which PPR site(s) are important for growth and viability, is still to be identified. We detected *in vitro* ZBTB43 binding at about one third of predicted Z-DNA sites, and *in vivo* binding at much less of those sites. ZBTB43 may select the most fragile sites and protects those from DSBs. Alternatively, ZBTB43 may select specific Z-DNA sequences in the germline that have evolved to be of value for regulatory purposes, by facilitating essential methylation at those sites.

We find that removing Z-DNA structures in the nucleus by ZBTB43 is specific to the germ cells in the fetal testis where it has relevance to genome integrity and DNA methylation. It will be interesting to find out how general these findings are. Is there scheduled remodeling of Z-DNA in other cell types, for example in the female germ line, and does it affect DNA methylation of PPRs there? Do other ZBTB proteins recognize Z-DNA, and do they have a function in remodeling Z-DNA in any cell type? Do they carry out their other biological functions by remodeling DNA structure? Does any ZBTB protein recognize the newly discovered eGZ motif [73] which forms at CGG trinucleotide repeats in the left-handed Z-DNA helix shape? Such finding would expand the scope of Z-DNA binding proteins beyond dinucleotide repeats to understanding trinucleotide repeat stability. Indeed, trinucleotide repeat stability is related to unusual DNA structures and may be epigenetically regulated [74–77].

Z-DNA is only one type of alternative DNA structure. Fragile sites in the genome are found at such sequences that can form at alternative DNA structures and are thought to break easily due to DNA structure formation at those sequences [52]. What removes other alternative DNA structures in the germ line? Is there scheduled remodeling of any other non-B DNA structures in the germ line or in the organism? Do other ZBTB proteins bind any other alternative DNA structures? Has the ZBTB family evolved to safeguard genome integrity by recognizing and remodeling different types of non-B-DNA structures? Several ZBTB family members have specific roles during T-cell development [20]. It will be interesting to find out whether the developmental function of ZBTBs involves remodeling alternative DNA structure.

Self-renewal in adult spermatogonia requires PLZF (ZBTB16) [78]. PLZF is also required for regulating the balance between self-renewal and differentiation in neurogenic, osteogenic, and hematopoetic progenitors [79]. It is not known, however, if PLZF is involved in DNA structure remodeling, and if the stem cell state requires keeping certain sequences in the B-DNA shape. One recent study suggests based on single cell transcriptomics and CRISPR screening data that ZBTB43 might be a potential actor in the cell self-renewal process in cancer [80]. Mechanistically, keeping the B-DNA structure may be important in the stem cell stages during development, as another transcription factor, ZSCAN4, retains nucleosomes at (CA)_n_ microsatellite repeats, ensuring that they remain in the B-DNA structure in ES cells [81]. It is interesting to note that apart from similarity to other ZBTB proteins, ZBTB43 shows 11% identity at the amino acid level with the ZSCAN4 genes, and they are classified in Ensembl [82] as ancient paralogs. The ZBTBs closest to ZBTB43 are ZBTB7a and ZBTB5 with 16% and 15% identity.

ZBTB43 affects *de novo* DNA methylation by remodeling Z-DNA to B-DNA. Another member of the ZBTB family, ZBTB24 is mutant in 30% of ICF2 syndrome, which is characterized by DNA hypomethylation at centromeric α-satellite and pericentromeric satellite-2/3 repeats and at other, mostly heterochromatic and late replicating parts of the genome [83]. ICF2 lymphocytes exhibit multiradial chromosomes, resulting from chromosomal breaks [84]. The primary cause of the abnormal chromosomes may be DNA hypomethylation at the pericentric regions [84]. Is it possible that ZBTB24 remodels secondary DNA structures to prevent ICF2? It is interesting to note that according to structural analysis, ZFs 5–7 of ZBTB24 are involved in DNA binding, but ZF4 is also involved in gene activation [47] and its mutation C383Y is causative in ICF2 [38,41]. Also, only 50% of ZBTB24 genomic binding sites occur at the CTGCCAGGACCT consensus [46,47], and we speculate that the other half may occur at DNA structures. Is it possible that ZF4 of ZBTB24 is important at those sites for binding secondary DNA structures? Does ZBTB24 or do other ZBTB proteins enable DNA methylation by remodeling DNA structures similar to ZBTB43? It can be expected that remodeling any secondary DNA structure to B-DNA will generate the substrate for enzymes that recognize DNA in the B-DNA shape.

**Figure 3.**
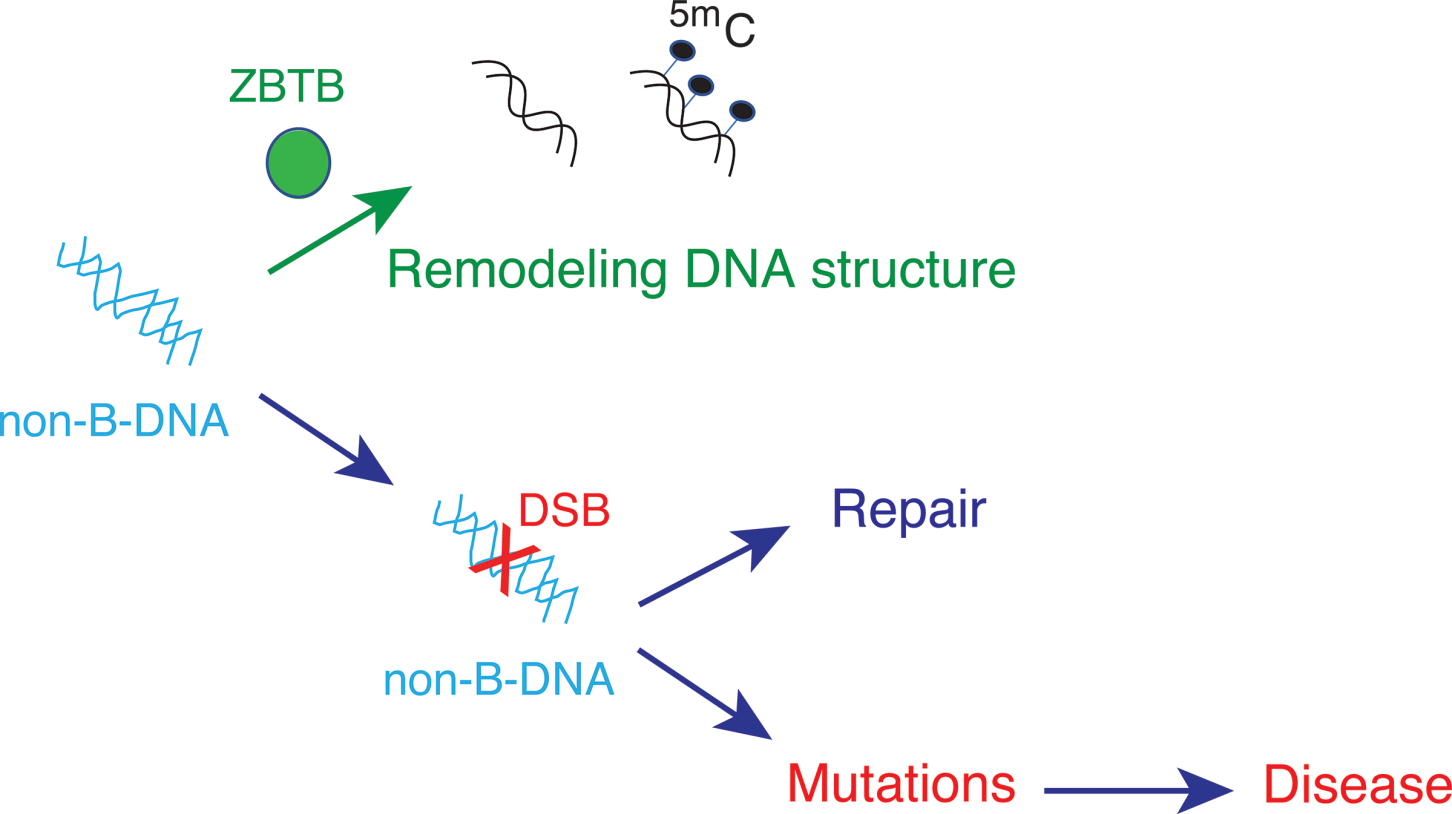
Preventing versus repairing double strand breaks at non-B DNA structures are two distinct possibilities for avoiding disease. The ZBTB family may have evolved to interfere with DSB formation, and remodeling of non-B DNA structures may occur in other cell types in addition to prospermatogonia. Other ZBTB proteins may also help to induce B-DNA and establish the normal DNA methylation patterns required for healthy cell function. The preventive mechanism of remodeling non-B DNA structures is distinct from other protective mechanisms that repair DSBs to avoid mutations and disease. This feature of ZBTB43 and potentially other ZBTBs could be considered in medicine.

DSBs have deadly consequences to causing mutations and diseases (Figure 3). Fragile sites share an important feature, the ability to form non-B DNA structures [52]. Recent years have seen concerted efforts toward understanding the mechanisms of how DSBs are generated and repaired, with clear implications to prevention and therapy of disease [53,63,72,85–87]. In addition to the mechanisms that repair DSBs at non-B-DNA fragile sites, is it possible that a general preventive mechanism has evolved as is exemplified by ZBTB43-mediated remodeling of Z-DNA to B-DNA? B-DNA could be generated from the dangerous non-B-DNA in specific cells, and this may or may not also affect DNA methylation. If the answer is yes, then such remodeling mechanism may be harnessed in prevention of human disease (Figure 3).

## Perspectives

- Remodeling of DNA structure not only coincides with the major wave of global epigenetic remodeling in the fetal male germline, but these layers of reprogramming are functionally connected: epigenetic remodeling depends on remodeling DNA structure. First example when Z-DNA structure can be genetically manipulated in an organism, revealing that DSBs occur at PPRs because of the Z-DNA structure and not simply the PPR DNA sequence.
- The germ line genome and epigenome integrity are protected by remodeling Z-DNA. The specific findings on Z-DNA remodeling by ZBTB43 in prospermatogonia may have more general implications to scheduled remodeling of other DNA structures, similar remodeling functions for other ZBTB proteins, and additional cell types.
- Follow-up studies will characterize the molecular mechanism, structural aspects, and dynamics of genome wide Z-DNA remodeling by ZBTB43 relative to DNA methylation at specific sites in prospermatogonia, and test whether ZBTB43 prevents mutations in the germ line. Future studies will test whether other ZBTB proteins have the ability to bind and change non-B DNA structures.

## Abbreviations

BTB: broad-complex, Tramtrack and Bric-à-brac
DNMT1: DNA methyltransferase 1
dpc: days post coitum
DSB: double strand breaks
ICF2: immunodeficiency, centromeric instability and facial anomalies 2
NHEJ: nonhomologous DNA end joining
PPR: purine/pyrimidine dinucleotide repeat
ZBTB: zinc finger domain containing
